# Application of protein purification methods for the enrichment of a cytotoxin from *Campylobacter jejuni*

**DOI:** 10.1186/1471-2180-12-303

**Published:** 2012-12-23

**Authors:** Xenia Gatsos, David L Steer, Thamradeen A Junaid, A Ian Smith, Ben Adler, M John Albert

**Affiliations:** 1 Australian Research Council Centre of Excellence in Structural and Functional Microbial Genomics, Monash University, Clayton, Victoria, Australia; 2 Department of Pathology, Faculty of Medicine, Kuwait University, Jabriya, Kuwait; 3 Department of Microbiology, Faculty of Medicine, Kuwait University, Jabriya, Kuwait

**Keywords:** *C. jejuni*, Cytotoxin, Biochemical methods, HPLC ion-exchange chromatography

## Abstract

**Background:**

*Campylobater jejuni*, a major foodborne diarrhoeal pathogen is reported to produce a number of cytotoxins of which only a cytolethal distending toxin (CDT) has been characterised so far. One or more additional cytotoxins other than CDT, including a Chinese hamster ovary (CHO) cell active, Vero cell inactive cytotoxin, may mediate inflammatory diarrhoea. Our objective was to develop a method to enrich and thus partially characterise this cytotoxin, as a pathway to the eventual identification and characterisation of the toxin.

**Results:**

A number of biochemical methods including cation- and anion-exchange chromatography were evaluated to enrich the cytotoxin from a cell lysate of a known cytotoxin-producing *C. jejuni*, C31. The cytotoxin in crude lysate was initially prepared by size-exclusion desalting and then subjected to high pressure liquid chromatography (HPLC) ion-exchange fractionation. One pooled fraction (pool B) was cytotoxic for CHO cells equivalent to crude toxin (tissue culture infectivity dose 50 [TCID_50_] of 1–2 μg/ml). The proteins of pool B were identified by mass spectrometry (MS) after separation by SDS-PAGE and trypsin digestion. Also, pool B was directly digested with trypsin and then subjected to liquid chromatography tandem mass spectrometry (LCMS) analysis for identification of lesser abundant proteins in the fraction. A total of 41 proteins were found in the fraction, which included enzymes involved in metabolic and transport functions. Eighteen non-cytoplasmic proteins including 2 major antigenic peptide proteins (PEB2 and PEB3) and 3 proteins of unknown function were also identified in the screen. Cytotoxicity in pool B was trypsin-sensitive indicating its protein nature. The cytotoxic activity was heat-stable to 50°C, and partially inactivated at 60-70°C. The pool B fraction also induced fluid accumulation in the adult rabbit ileal loop assay with cytotoxicity for mucosa confirming the presence of the cytotoxin.

**Conclusions:**

We report the enrichment and partial purification of *C. jejuni* cytotoxin by HPLC ion-exchange chromatography. Further purification may be achieved using additional complementary chromatographic techniques. A short-list of six candidate cytotoxin proteins was identified using an LCMS screen of pool B. Successful isolation of the cytotoxin will initiate steps for the determination of the role of this cytotoxin in the pathogenesis of *C. jejuni* diarrhoea.

## Background

*Campylobacter jeuni* is a foodborne pathogen and a major cause of bacterial diarrhoea worldwide [1], yet its pathogenicity is poorly understood. The virulence attributes of *C. jejuni* include cell culture adherence and invasion, flagella and motility, iron-acquisition capability and toxin production [2]. Known toxins include a cytolethal distending toxin (CDT), a cholera toxin-like enterotoxin (CTLT), and a number of cytotoxins [3]. However, only the genes encoding the CDT have been identified so far [4]. There is uncertainty on the production of CTLT by *C. jejuni*. Our recent work indicated that the major outer membrane protein (MOMP-PorA) of *C. jejuni* cross-reacts with cholera toxin (CT) which would likely have misled investigators that *C. jejuni* produces a CTLT [5]. It is believed that the cytotoxin(s) may mediate inflammatory diarrhoea that is characteristic of infection in individuals in developed countries [6]. One major cytotoxin is a protein-sized molecule that is active on a number of cell lines such as HeLa and Chinese hamster ovary (CHO), but is inactive on Vero cells [3]. A previous report claimed that the MOMP of *C. jejuni* was responsible for cytotoxicity on mammalian cells [7]. However, in our previous work, the expressed PorA protein from the cloned gene of a cytotoxin-producing *C. jejuni* strain did not have cytotoxic activity for mammalian cells and was also devoid of diarrhoeagenic activity in an animal model of infection [8]. In our continuing efforts to characterise this unknown cytotoxin, we investigated a series of chromatographic methods to enrich the cytotoxin from a cytotoxic *C. jejuni* strain. Using previously established methods of detection as well as further modifications to these protocols, we have attempted to isolate and purify the cytotoxin. The results of further characterisation studies confirm that the likely cytotoxin candidate is a protein. The results are reported in this communication.

## Results and discussion

### Cytotoxicity assay

**Figure 1:**
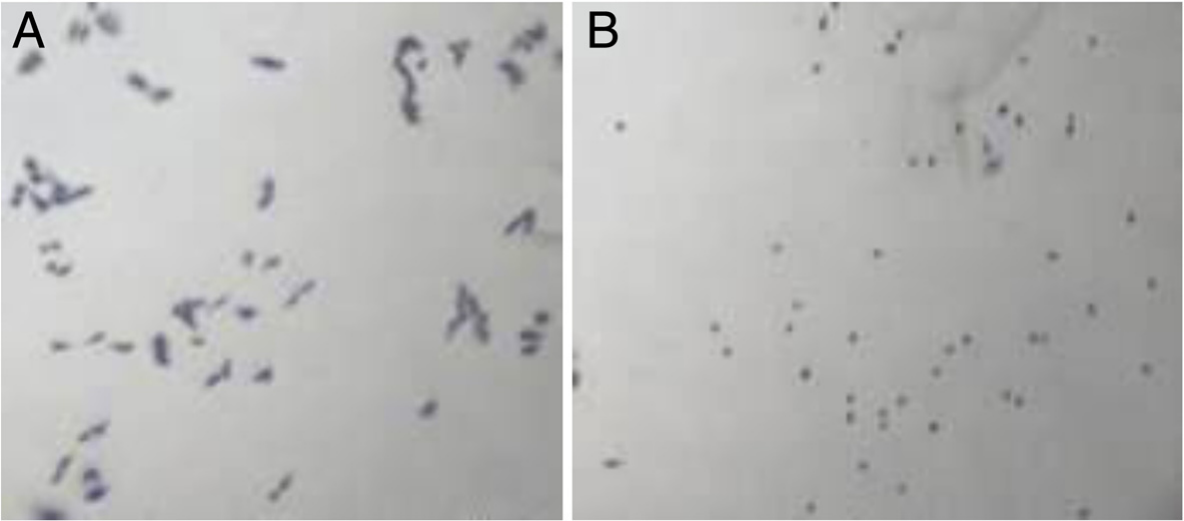
**Effect of *C. jejuni *crude protein extract on CHO cells.** Panel **A** shows appearance of normal cells and panel **B** shows cytotoxic effect on cells upon inoculation with 4 μg crude protein extract from *C. jejuni* C31 strain. Magnification x 100.

In this study, we have developed a methodology to detect and purify the toxin potentially involved in the diarrhoeagenic activity of *C. jejuni,* C31 strain. To detect and quantify cytotoxic activity during purification, we used an activity assay based on the lethal effects of the toxin on CHO cells. The TCID_50_ of C31 strain for CHO cells was similar at 1–2 μg for a freshly prepared protein extract as well as a reconstituted form of the lyophilised extract as estimated by the visual method by direct microscopic observation of cytotoxic effect on cells [8] or by the indirect methyl thiazol tetrazolium (MTT) method by spectrophotometric measurement of formazin [9]. The cytotoxic effect of C31 toxin on CHO cells is shown in Figure 1. The extract was devoid of any cytotoxic effect when tested on Vero cells as described previously [8].

### Extract fractionation and cytotoxin purification

We sought to employ a series of chromatographic methods to enrich and isolate the cytotoxin as a prelude to proteomic analysis to identify it. The key to this strategy was the CHO cell cytotoxicity assay to monitor the presence of the cytotoxin in various fractions obtained by our purification techniques. We initially exposed the protein extract to the various buffers and conditions likely encountered throughout the course of the enrichment procedure to determine which conditions were suitable for maintaining the stability of the cytotoxin (data not shown). In these initial tests, we found that activity was maintained in buffers containing up to 1 M NaCl, allowing the use of ion-exchange and size-exclusion chromatography. We also found that exposure to low pH and organic solvents such as acetonitrile did not reduce activity, thereby allowing the expansion of our enrichment procedures to the use of reversed phase chromatography. In addition to classical chromatography, we also used OFFGEL electrophoresis, a recently developed technique, separating proteins based on their isoelectric point into discrete fractions; however after no activity was recovered in these experiments (data not shown),we then focused on the use of classical chromatography.

**Figure 2:**
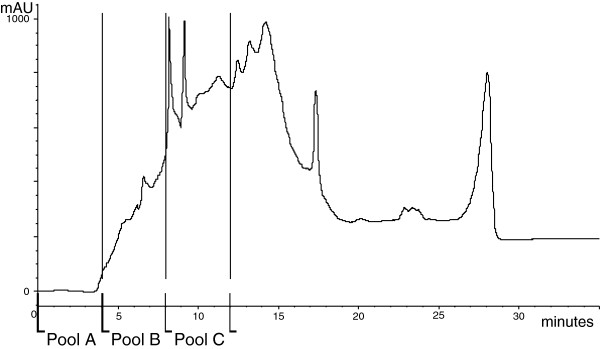
**HPLC trace of protein elution with increasing salt concentration.** The trace shows the UV absorbance as milli-absorbance units (mAU) by the eluting proteins on the y axis against time on the x axis. The gradient was run from 0 to 1 M NaCl over 30 minutes. Collected pooled fractions are shown.

After sample preparation using size- exclusion based desalting, we performed cation- exchange chromatography collecting individual fractions of which every 4 fractions were pooled. Table 1 shows the results of the first three pooled fractions including protein recovery in comparison to the starting protein extract. Figure 2 shows an example HPLC trace of the protein elution profile from the ion-exchange column with increasing salt concentration with the pooled collected fractions overlaid. Pool A essentially consists of the first 4 minutes where no UV absorbance was observed, pool B consists of the weakly charged early eluting proteins, as seen by the rise in UV absorbance. Cytotoxic activity was also observed in pool B and this fraction was thus used for further analysis. Pool C fractions consisting of fractions between 8 and 12 minutes contained some high abundance proteins as observed by the large peaks eluting at 8 and 9 minutes.

**Table 1:** Cytotoxic activity and recovered protein concentration of the HPLC ion- exchange fraction pools of *C. jejuni* extract

**Assayed sample**	**Fractions pooled**	**Cytotoxic activity observed**	**Protein concentration (mg/ml)**
Untreated extract	Not applicable	Yes	3.55
Pool A, 0–4 mins	1-4	No	0.0
Pool B, 4–8 mins	5-9	Yes	1.16
Pool C, 8–12 mins	10-14	No	1.65

### Stability of fraction B cytotoxin to protease digestion and heat treatment

**Figure 3:**
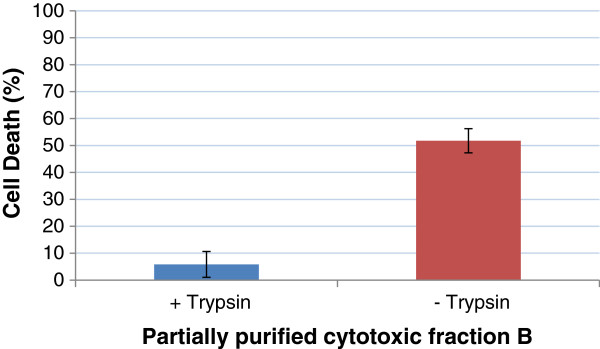
**Stability of cytotoxic activity of pool B to trypsin digestion.** Pool B (2 μg/ml) was treated with and without 125 μg/ml trypsin. The samples were then incubated with CHO cells overnight. Percent CHO cell death was determined using the MTT assay. Experiment was performed in triplicate, error bars represent standard error of mean (n = 3).

Pool B was used for further analysis as it contained the highest level of cytotoxic activity. To further characterise the toxin and confirm that it is a protein, we examined the effect of protease digestion on cytotoxin activity. Incubation with trypsin reduced the toxicity of the partially purified cytotoxin for CHO cells (Figure 3). This finding likely reflects that the cytotoxic component of the preparation is a protein. The partially purified cytotoxin was subjected to incubation at elevated temperatures and the observed cytotoxic activity was compared with the unincubated control samples (22°C) and we found that activity was unaffected at 50°C, but was reduced at higher temperatures (90% active at 60°C and 70% active at 70°C) suggesting that the cytotoxin is relatively heat- stable (data not shown).

### Cytotoxin activity confirmation *in vivo*

**Figure 4:**
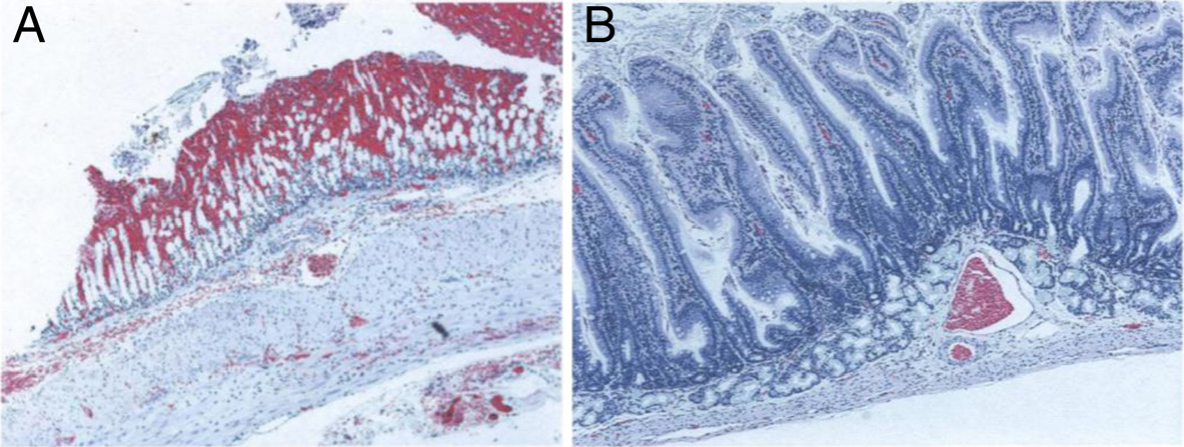
**Histopathology of the adult rabbit intestinal loops inoculated with pool B fraction.** In panel **A**, the loop was injected with pool B fraction and stained with eosin and haematoxylin. The mucosa shows oedema, inflammation and necrosis. In panel **B**, the loop was injected with Sorenson’s buffer (negative control) and stained with eosin and haematoxylin. The mucosa appears normal. (Magnification x 50 for both sections).

To further confirm that the activity isolated in pool B was due to the cytotoxin, the rabbit ileal loop assay was employed to detect the presence of diarrhoeagenic activity. The positive *E. coli* control induced a large amount of fluid (mean volume [ml] to length of loop [cm] ratio was 2.0), *C. jejuni* C31 whole cell lysate and the pool B fraction induced moderate amounts of fluid (mean volume/length ratio was 0.4 for C31 lysate and 0.8 for pool B fraction). The negative control, Sorensen’s buffer, and fractions A and C did not induce any fluid secretion. On histopathology, the intestinal loops injected with the pool B fraction or *C. jejuni* C31 whole lysate showed oedema, congestion, haemorrhagic necrosis and inflammation of the mucosa (Figure 4A), whereas the loops injected with Sorenson’s buffer and fractions A and C appeared normal (Figure 4B). The fluid accumulation and mucosal changes are similar to the findings of a previous study using *C. jejuni* isolates from patients with inflammatory diarrhoea [10]. This shows that fluid secretion and mucosal inflammatory changes are mediated by the cytotoxic pool B. Previous studies with crude lysate of C31 showed fluid accumulation in the rabbit ileal loop assay [8].

### Proteomic identification of pool B

As the potential cytotoxin co-purified in pool B exhibited cytotoxic activity, pool B was subjected to MS analysis for protein identification. This was done using a combination of SDS- PAGE and LCMS as well as the LCMS analysis of a complete trypsin digest of pool B to identify any lesser abundant proteins. Forty-one proteins were isolated and identified from fraction pool B (Table 2). Of these, 23 were predicted to reside in the cytoplasm and most have conserved functions in metabolic processes, redox reactions, transcription/translation control and protein processing and turnover. BLAST searches were performed to identify homologues or conserved domains in other species to assist in defining a role for potential candidate cytotoxins. The online program PSORTb was used to predict localisation of the identified proteins. One uncharacterised protein, A7H5H8 contains a cupin-2 domain which is characterised by the presence of a double stranded beta helix. The function of this protein and domain are unknown, although as it is predicted to reside in the cytoplasm, it is unlikely to be a cytotoxin candidate.

**Table 2:** Combined dataset of full list of proteins identified in pool B after direct trypsin digestion and SDS-PAGE separation

**Accession number**	**Full identification name**	**Biological function known or inferred**	**Localisation**	**Mascot score**	**Total matches**	**Peptides matched**	**Sequence coverage (%)**
A1VXN0 (Cj0146c)*	Thioredoxin reductase	Oxidation-reduction process	Cytoplasm	353	10	4	20
A1VXN1 (cj0147c)	Thioredoxin	Metabolic	Cytoplasm	1931	41	4	56
A1VXS1 (Cj0193c)	Trigger factor	Cell division	Cytoplasm	33	1	1	2
				62^c^	4^c^	3^c^	6^c^
A1VXZ3 (Cj0269)	Branched-chain amino acid aminotransferase	Metabolic	Cytoplasm	146	10	3	17
A1VY51 (Cj0332c)	Nucleoside diphosphate kinase	Nucleotide metabolism	Cytoplasm	80	2	1	16
A1VY53 (Cj0334)	Antioxidant, AhpC/Tsa family	Cell redox homeostasis	Cytoplasm	1762	62	6	41
				90^c^	5^c^	5^c^	33^c^
A1VYF1 (Cj0434)	2,3-bisphosphoglycerate-independent phosphoglycerate	Gluconeogenesis	Cytoplasm	70	6	3	6
				50^c^	4^c^	4^c^	9^c^
A1VYI6 (Cj0470)	Elongation factor Tu (tuf)	Protein biosynthesis	Cytoplasm	94	7	3	10
				53^c^	4^c^	3^c^	10^c^
A1VYJ5 (Cj0479)	DNA-directed RNA polymerase subunit beta	Transcription	Cytoplasm	48	5	1	0
A1VYM1 (Cj0509c)	ATP-dependent chaperone protein ClpB	Metabolic	Cytoplasm	110	6	2	3
				100^c^	6^c^	5^c^	5^c^
A1VZ00 (Cj0640c)	Aspartyl-tRNA synthetase (aspS)	Protein biosynthesis	Cytoplasm	44	3	2	6
A1VZH9 (Cj0835c)	Aconitate hydratase	Metabolic	Cytoplasm	97	3	2	3
				127^c^	7^c^	6^c^	11^c^
A1VZR2 (Cj0929)	Cytosol aminopeptidase family protein	Proteolysis	Cytoplasm	68	3	1	2
				39^c^	3^c^	3^c^	5^c^
A1W057 (Cj1070)	30S ribosomal protein S6	Translation	Cytoplasm	226	8	1	8
A1W0G5 (Cj1181c)	Elongation factor Ts (tsf)	Protein biosynthesis	Cytoplasm	201	5	2	10
A1W0K3 (Cj1220)	10 kDa chaperonin (groS)	Protein folding	Cytoplasm	689	37	4	47
A1W0K4 (Cj1221)	60 kDa chaperonin (groL)	Protein folding	Cytoplasm	505	27	7	19
				179^c^	6^c^	5^c^	13^c^
A8FKV4 (Cj0531)	Isocitrate dehydrogenase, NADP-dependent	Metabolic	Cytoplasm	38	7	3	6
				355^c^	14^c^	12^c^	17^c^
A8FNK5 (Cj1545c)	MdaB-like protein	Oxidation-reduction process	Cytoplasm	81	3	2	12
A1VZC7 (Cj0779)	Thiol peroxidase	Oxidation-reduction process	Cytoplasm / periplasm	679	14	4	37
A8FKM7 (Cj0440c)	Putative transcriptional regulator	Transcription	Cytoplasm^a^	82	3	3	12
A1VXI8 (Cj0105)	ATP synthase subunit alpha	ATP synthesis	Cytoplasmic membrane	84	3	2	9
A1W0Q1 (Cj1266c)	Quinone-reactive Ni/Fe-hydrogenase, large subunit (hydB)	Oxidation-reduction process	Cytoplasmic membrane	200	7	3	9
A1W0V5 (cj1338c)	Flagellin B	Flagellar motility	Extracellular	2023	48	8	23
				608^c^	25^c^	13^c^	34^c^
A1VY12 (cj0289c)	Major antigenic peptide PEB3	Transport	Non-cytoplasmic	173	4	2	11
				304^c^	8^c^	6^c^	27^c^
A1VZC6 (cj0778)	Major antigenic peptide PEB2	Transport	Non-cytoplasmic	233	9	3	27
				159^c^	8^c^	6^c^	30^c^
A1VZK9 (Cj0866)	Arylsulfate sulfotransferase, degenerate	Transferase	Non-cytoplasmic	358	10	4	8
A1VZY6 (cj0998c)	Putative periplasmic protein	Unknown	Non-cytoplasmic	597	19	3	25
A1W0Q2(cj1240c)	Putative periplasmic protein	Protein binding	Non-cytoplasmic	151	11	3	13
A8FKY2 (Cj0559)	Putative pyridine nucleotide-disulphide oxidoreductase	Oxidation-reduction process	Non-cytoplasmic	200	7	3	9
				292^c^	17^c^	13^c^	49^c^
A8FLP3 (cj0834c)	Putative uncharacterised protein	Protein-protein interaction	Non-cytoplasmic ^a^	155^c^	8^c^	7^c^	19^c^
A8FKK7 (Cj0420)	Putative uncharacterised protein	Lipid binding protein	Non-cytoplasmic, likely periplasmic	331	12	2	14
A1VYD4 (Cj0414)	Putative oxidoreductase subunit	Oxidation-reduction process	Non-cytoplasmic^b^	581	11	3	26
A1VXJ7 (cj0114)	Putative periplasmic protein	Protein binding	Outer membrane	174	5	1	4
A1VXA8 (Cj0029)*	L-asparaginase	Metabolic	Periplasm	139	3	1	4
				63^c^	3^c^	3^c^	14^c^
A1VXB4	Gamma-glutamyltransferase	Transferase	Periplasm	575	18	6	14
				303^c^	23^c^	11^c^	19^c^
A1VXC2 (Cj0037c)	Putative cytochrome C	Electron carrier activity	Periplasm	46	1	1	5
A1VXM7 (Cj0143c)	Putative periplasmic solute binding protein for ABC transporter system	Metal ion binding	Periplasm	36	1	1	4
A1VY79 (Cj0358)	Putative cytochrome C551 peroxidase	Peroxidase	Periplasm	627	33	3	13
				283^c^	22^c^	13^c^	38^c^
A1W0L1 (Cj1228c)	Serine protease	Proteolysis	Periplasm	239	11	8	31
A8FKK2 (Cj0415)	Putative GMC oxidoreductase subunit	Unknown	Unknown	1830	71	16	38
				286^c^	15^c^	13^c^	28^c^

*Gene designation refers to the best match identified in *Campylobacter jejuni* NCTC 11168 (where present).

^a^ The programme signal was used for protein localisation prediction; non-cytoplasmic localisation refers to proteins whose localisations are unknown, but a cytoplasmic localisation is ruled out as a N-terminal signalling sequence is present.

^b^ The uniprot annotation was used for protein localisation prediction.

^c^ The identification data obtained from the gel based analysis.

Prediction of protein localisation was determined using the program PSORTb, and table listings are sorted by localisation.

Of the 18 non-cytoplasmic proteins identified, 7 are conserved amongst the proteobacteria and have roles in oxidation/reduction processes. Other conserved proteins are involved in protein synthesis and turnover (A1W0L1 and A1VYJ3), metabolism (A1VXA8, A1VXB4 and A1VZK9) and ATP synthesis (A1VX18). Of the remaining proteins predicted to be non-cytoplasmic, 3 are structural proteins involved in flagella biosynthesis, and are unlikely to be involved in cytotoxin biosynthesis or activity. The remaining proteins are predicted to have roles in protein-protein interactions or are involved in binding and transport of lipids (A8FKK7) or cations (A1VXM7).

A short list of six potential cytotoxin candidates is summarised in Table 3. PEB3 (A1VY12) was identified in the pool, and this protein has been previously characterised as a glycoprotein and adhesion protein involved in transport of phosphate-containing molecules [11]. PEB2 (A1VZC6), a major antigenic peptide of *C. jejuni* on the other hand, is a protein of unknown function which contains a similar signal sequence to PEB3 suggesting similar localisation [12]. It is conserved in *C. jejuni* and *C. coli* and BLAST hits return with matches to the accessory colonisation factor protein (acfC) of *Vibrio cholerae* (34% identical residues/53% positive residues) and a “Conserved Domain Search” on NCBI matched to domains involved in extracellular solute binding and transport systems. Based on these inferences, it is unlikely to be the cytotoxin of interest, although further study of this protein is warranted.

**Table 3:** Short-list of potential cytotoxin candidates identified from LCMS screen of pool B

**Accession number**	**Full identification name**	**Biological function known or inferred**	**localisation**	**Size (kDa)**
A1VY12 (cj0289c)*	Major antigenic peptide PEB3	Transport	Non-cytoplasmic	27.5
A1VZC6 (cj0778)	Major antigenic peptide PEB2	Transport	Non-cytoplasmic	27.0
A8FLP3 (cj0834c)	Putative uncharacterised protein	Protein-protein interaction	Non-cytoplasmic ^a^(signalP)	46.7
A1W0M3 (cj1240c)	Putative periplasmic protein	Protein binding	Non-cytoplasmic	23.0
A1VZY6 (cj0998c)	Putative periplasmic protein	Unknown	Non-cytoplasmic	20.5
A1VXJ7 (cj0114)	Putative periplasmic protein	Protein binding	Outer membrane	35.4

*Gene designation refers to the best match identified in *Campylobacter jejuni* NCTC 11168.

^a^ Protein localisation prediction was determined using the program signalP.

Prediction of protein localisation was determined using the program PSORTb.

Proteins A1W0M3 and A1VZY6 are hypothetical proteins and potential candidates for the cytotoxin, although their predicted sizes (23.0 kDa and 20.5 kDa) are relatively smaller than the high molecular weight cytotoxin previously characterised [3].

One prospective cytotoxin candidate (A1VXJ7), a 315 amino acid residue protein is a TPR family protein which indicates that it is involved in protein:protein interactions (residues 226–265). A tol/pal/ybgF domain at its C-terminus (residues 235–315) is also present. Proteins with this domain are required for stabilisation of the outer membrane of Gram-negative bacteria. No hypothetical functions or domains could be located to the N-terminus (residues 1–225) of this protein. Perhaps, the C-terminal portion allows direct contact with a protein receptor on the host cell, and the N-terminus contains a cytotoxin function.

The protein most likely to be involved in cytotoxic function is A8FLP3, a 412 amino acid residue protein which contains ankyrin repeat domains near its C-terminus (residues 180–375). A BLAST search identified mainly *C. jejuni* and *C. coli* strains with a similar protein, and only the ankyrin repeat domain returned hits to ankyrin repeat domains of eukaryotes.

Ankyrin repeat domains are traditionally associated with eukaryotic cellular functions, but more recently many intracellular pathogens have been discovered to secrete (through their T4SS) ankyrin repeat-domain containing proteins into their hosts which act to subvert the eukaryotic host functions and allow for their survival (reviewed in reference [13]). It has been suggested that cytotoxin induced CHO cell rounding could involve the reorganisation/inhibition of the cytoskeletal network of the cell [14], and several ankyrin-repeat containing proteins of *Legionella pneumophila* have the ability to interfere with microtubule-dependent vesicle transport [15]. Perhaps, this *C. jejuni* ankyrin repeat protein may also interfere with the cytoskeletal network of CHO cells. Further characterisation of this protein is required to identify its function.

In this study, we have sought to isolate the protein responsible for cytotoxic activity. We have successfully developed a protocol to extract proteins from the lysate of a suspension of cells retaining the activity of this protein. We have partially purified the protein possessing cytotoxic activity through the development of a protocol for the preparation of the protein extract followed by fractionation by HPLC using ion- exchange chromatography. This protocol resulted in the partial purification and enrichment of the active protein. Further experiments will be required to further purify the protein using chromatographic techniques additional to cation- exchange, such as reversed phase chromatography, although chromatography alone may not be sufficient to achieve absolute purity. This however, may not be necessary as from the proteins identified in the purified fraction, we could establish a short list of candidate proteins and through additional experiments, such as mutant knockout studies, confirm the identity of the cytotoxic protein. Interestingly, the pooled fraction B did not contain the major outer membrane protein, PorA. This suggests that PorA is not contributing to cytotoxic activity of fraction B [8]. We have shown that the fraction pool B, was shown to induce fluid secretion in the rabbit intestinal loop assay causing cytotoxic damage to the mucosa. We further characterised the toxin showing that, although the toxin is stable at elevated temperatures (50°C), activity was reduced with increasing temperature and activity was lost after trypsin digestion, confirming that toxic activity is a result of a specific protein function. However, our data do not exclude the possibility that cytotoxic effects may be mediated by a mixture of proteins.

Guerrant et al. [16] reported that the cytotoxin is a periplasmic protein as it can be extracted by polymyxin B. However, in our hands, polymyxin B interfered with the CHO cell assay, as it produced cytotoxic effects similar to the *C. jejuni* cytotoxin (unpublished data).

## Conclusions

Even though *C. jejuni* is a major foodborne diarrhoeal pathogen causing significant morbidity and mortality, its pathogenesis is poorly understood. It is important to purify and characterise its major cytotoxin to define its role in pathogenesis. We have succeeded in developing a method (HPLC ion-exchange purification method) for enriching and partially purifying the cytotoxin. Further studies are required for a complete purification of the cytotoxin.

The cytotoxin may be highly active at very low concentrations, low enough to remain undetected by our current proteomics identification procedures, removing most of the contaminating proteins via sub-fractionation of the cell should increase the chances of isolating and identifying this cytotoxin. One other option is to purify the supernatant of broth culture of *C. jejuni*, although given its fastidious nature and slow growth rate, high levels of active cytotoxin may be difficult to purify from the supernatant.

In this paper, we present preliminary data in our attempt to isolate, purify and identify the protein involved in cytotoxic activity of *C. jejuni.* We have employed an activity assay based on the lethal effects of the toxin on CHO cells to rapidly screen for activity and used this assay to screen chromatographic fractions to locate the presence of the active protein. We have been unable to unequivocally identify the protein as the sample remains too complex although we have identified some previously uncharacterised non-cytoplasmic proteins which with further experimentation potentially may be attributable to the cytotoxin. We will attempt further isolation of the protein so that we are then able to sequence and identify the protein. The activity of the toxin containing fraction was validated by performing the rabbit ileal loop assay.

## Methods

### Preparation of the cytotoxin and its detection

The reference cytotoxin-positive *C. jejuni* strain, C31 used in our previous study was used in this study [8]. The organism was grown on 7% sheep blood agar in a microaerobic atmosphere generated with BBL gaspak (Becton Dickinson, Sparks, MD, USA) in a jar with catalyst at 42°C for 48 h. The bacterial growth was suspended in phosphate-buffered saline (PBS, pH, 7.2) to McFarland’s opacity of 10 (equivalent to 3 X 10^9^ cells). The cytotoxin was released by sonication of bacterial cells in a sonicator (Soniprep 150 MSE, London,United Kingdom) at 10 amplitude microns for 20 min. The lysed cell suspension was centrifuged at 12,000 rpm at 4°C for 15 min in a Beckman centrifuge (J2-M1 with JA 20 rotor) and the supernatant was filtered through a sterile Techno Plastic Products (TPP, Zollstrasse, Switzerland) membrane filter (0.22 μm pore diameter). The fresh filtrate and the filtrate after freeze-drying were tested on Chinese hamster ovary (CHO) cells as described previously [8]. Doubling serial dilutions of the toxin in F-12 medium (Gibco, Paisly, United Kingdom) with a starting dilution of 1:2 were tested. Cytotoxic activity was characterised by cell rounding, granulation and eventual sloughing. The toxin titre was expressed as tissue culture infectivity 50 (TCID_50_) dose, the concentration of the toxin that caused cytotoxicity in 50% of the monolayer. In some instances, we used the methyl thiazol tetrazolium (MTT) assay [9] to quantitate cytotoxin activity. Metabolically active CHO cells are able to reduce the formazin present in the MTT reagent resulting in a colour change, allowing spectrophotometric quantitation of the activity of the cytotoxin. Results were calculated as a percentage of cell death when compared to a control using the equation: 1-(test well/control well) ×100. The experiment was performed in biological triplicates. The Students t-test was used for statistical analysis; a P value of ≤0.05 was considered significant.

The effect of cytotoxin on Vero cell was investigated as described previously [8].

### Fractionation of cytotoxin with OFFGEL electrophoresis

Fractionation was done using the Agilent OFFGEL Fractionator (Agilent Technologies, Santa Clara, CA, USA). The toxin preparation (freeze-dried and reconstituted in distilled water) was desalted using the 2D cleanup kit according to manufacturer’s instructions (GE Healthcare Biosciences, AB, Uppsala, Sweden) and the precipitated protein was reconstituted in the OFFGEL running buffer. The sample was then fractionated using a 13 cm, 3–10 pH range IPG strip collecting 12 fractions according to the manufacturer’s instructions.

### Sample preparation for HPLC ion- exchange fractionation

Typically 100 mg of extract was reconstituted in 100 μl water, centrifuged to remove insoluble material and desalted using size-exclusion (SE) based device, the Zeba Spin desalting column (Pierce, Rockford, IL, USA) according to manufacturer’s instructions.

### HPLC ion- exchange fractionation

HPLC purification was performed on an 1100 series microbore HPLC (Agilent technologies). The preparation obtained from the SE spin column was diluted to 500 μl in Soreneson’s buffer, pH 7.4 (Buffer A). Samples were injected onto an ion-exchange column Mono Q HR 5/5 (GE Healthcare Biosciences) with buffer A at a flow rate of 150 μl/minute. The proteins were eluted over a 30-minute linear gradient to 100% B (Sorenson’s buffer, pH 7.4, 1 M NaCl). Fractions were collected at 1 minute intervals and every four fractions were pooled. Pooled fractions were concentrated to 500 μl using nanosep 10 k cutoff centrifugal device (Pall Life Sciences, MI, USA). In preparation for the MTT assay, the resultant fractions were diluted to 2 ml volumes with Sorenson’s buffer.

### Mass spectrometry (MS)

Trypsin digests on excised gel bands were performed in a solution of 20 mM ammonium bicarbonate containing 0.5 μg trypsin (Promega corporation, Madison, WI, USA) and then analysed directly by LCMS as outlined below. Trypsin digests on the pool B fraction directly were performed in a solution of 20 mM ammonium bicarbonate containing 10 μg trypsin (Promega corporation) and then the resultant digested peptides were fractionated by 12 salt plug elutions ranging from 2 mM to 500 mM NaCl from a SCX TopTip (Glygen, Columbia, MD, USA) according to manufacturer’s instruction. Both digest protocols were incubated at 37°C for 12 hours. Tryptic digests were analysed by LC-MS/MS using the HCT ULTRA ion trap mass spectrometer (Bruker Daltonics, Bremen, Germany) coupled online with a 1200 series capillary HPLC (Agilent technologies). Samples were injected onto a zorbax 300SB reversed phase column with buffer A (5% acetonitrile 0.1% formic acid) at a flow rate of 10 μl/minute. The peptides were eluted over a 30-minute gradient to 55% B (90% acetonitrile 0.1% formic acid). The eluant was nebulised and ionised using the Bruker electrospray source using the low flow electrospray needle with a capillary voltage of 4000 V dry gas at 300°C, flow rate of 8 l/minute and nebuliser gas pressure at 1500 mbar. Peptides were selected for MSMS analysis in autoMSn mode with smart parameter settings selected and active exclusion released after 1 minute.

Data from LCMSMS runs were processed using Data Analysis 3.4 (Bruker Daltonics) and were exported in Mascot generic file format (*.mgf) and searched against an in-house database comprised of *C. jejuni* FASTA format genomes downloaded from the National Center for Biotechnology Information (NCBI) FTP site using the MASCOT search engine (version 2.1, Matrix Science Inc., London, United Kingdom) using MUDPIT scoring. The mgf files from the salt plug elutions were combined into a single mgf file. The following search parameters were used: missed cleavages, 1; peptide mass tolerance, ± 0.4 Da; peptide fragment tolerance, ± 0.2 Da; peptide charge, 2+ and 3+; fixed modifications, carbamidomethyl; variable modification, oxidation (Met).

### Stability of cytotoxin to protease digestion

The cytotoxin in pool B fraction was treated with trypsin (125 μg/ml) (Sigma, St. Louis, MO, USA) for 4 h at 37°C. The trypsin was inactivated by the addition of 125 μg/ml soybean trypsin inhibitor (Sigma). One hundred microliters of treated pool B fractions at a concentration of 2 μg/ml were added to a CHO cell monolayer in a microtitre plate. The MTT assay [9] for cytotoxicity was performed after a 24 h incubation period. Control samples consisted of PBS and trypsin inhibitor and were used in the calculation of percent cell death. The experiment was performed in triplicate and the Students t-test used to determine statistical significance.

### Heat stability of the cytotoxin

Triplicate samples of the cytotoxin in pool B fraction extract were incubated at 50°C, 60°C, or 70°C, for 30 min. The MTT assay was then performed for cytotoxicity [9].

### Rabbit ileal loop assay of pool B fraction for diarrhoeagenic activity

The ability of pool B fraction to induce fluid accumulation and cause inflammatory changes in the mucosa was studied in the adult rabbit ileal loop assay [10]. The concentration of the fraction B tested was 0.2 mg/ml, and 1.0 ml of the fraction was inoculated into single small intestinal loops (approximately 10 cm long) of two adult rabbits. A similar concentration of fraction A and fraction C was also tested. The negative control loop was inoculated with Sorensen’s buffer (diluent used to dissolve the toxin) and the positive control loops were inoculated with a whole lysate of *C. jejuni* C31 strain [8] or a broth culture of enterotoxigenic *Escherichia coli* (strain H10407). After 20 h of inoculation, the rabbits were sacrificed, the characteristics and amount of fluid accumulated noted and tissue sections taken in neutral formal saline for processing for histopathology by staining with eosin and haematoxylin stain. Coded slides were examined by a histopathologist.

The procedures involving animals were according to the guidelines for animal research of the Health Sciences Centre, Kuwait University.

## Abbreviations

CHO cell: Chinese hamster ovary cell; CDT: Cytolethal distending toxin; CTLT: Cholera toxin-like enterotoxin; MOMP: Major outer membrane protein; MTT method: Methyl thiazol tetrazolium method; HPLC: High pressure liquid chromatography; MS: Mass spectrometry; LCMS: Liquid chromatography tandem mass spectrometry; SDS-PAGE: Sodium dodecyl sulphate-polyacrylamide gel electrophoresis.

## Competing interests

None of the authors has competing interests.

## Authors' contributions

MJA, BA and AIS conceived the study. In addition, MJA carried out the rabbit ileal loop assay. DLS performed the cytotoxin purification methods. XG performed the assays for the cytotoxin. TAJ carried out the histopathological studies. All authors participated in the writing of the manuscript and read and approved the final manuscript.

## Authors’ information

MJA and TAJ are Professors of Microbiology and Pathology respectively at the Faculty of Medicine, Kuwait University, Kuwait. BA and IAS are Professors of Microbiology and Biochemistry respectively at Monash University, Australia. XG is a Post-doctoral Fellow in the Department of Microbiology and DLS is Research Manager in the Department of Biochemistry, both at Monash University, Australia.
